# Dark White Matter: Evidence of a Peri-Ictal MRI Sign from a Systematic Review

**DOI:** 10.3390/jcm14134607

**Published:** 2025-06-29

**Authors:** Giuseppe Magro, Olindo Di Benedetto, Antonio Di Renzo, Emanuele Tinelli

**Affiliations:** 1 Department of Neurology, Lamezia Terme Hospital, 88046 Catanzaro, Italy; 2 Neuroradiology Department, ‘Magna Graecia’ University, 88100 Catanzaro, Italy

**Keywords:** dark white matter, subcortical hypointensity on FLAIR/T2, FLAIR hypointensity, brain MRI, magnetic resonance imaging, seizures, peri-ictal MRI abnormalities (PMAs), seizure-induced reversible MRI abnormalities (SRMA), periictal imaging

## Abstract

The radiological finding of Dark White Matter (DWM)—characteristic diffuse subcortical white matter hypointensity on T2/FLAIR sequences—is underrecognized, but has important clinical implications. Recent systematic evidence shows that over 60% of previously published cases showed seizures in association with DWM findings—it is also particularly predictive of the underlying etiology, particularly non-ketotic hyperglycemic hyperosmolar state (NKH). Based on our previous work, we reinterpret the data, focusing only on patients with seizures and DWM, to summarize the most essential and distinguishing features of these patients. Both cortical and subcortical abnormalities in DWM are more frequently associated with anti-MOG encephalitis. DWM with or without cortical involvement is more commonly found in NKH among patients with seizures. This updated systematic review will describe the proposed pathophysiological mechanisms, clinical associations, and implications for DWM in patients with seizures, and highlight how early recognition of DWM may allow for targeted diagnostic strategies and treatment options. We expanded our previous search with details regarding seizure features, our results show that DWM is associated with repetitive seizures and Status Epilepticus (both convulsive and non), in line with other peri-ictal MRI abnormalities associated with prolonged seizure activity. DWM-associated seizures are mostly focal, rather than generalized. Moreover, the high percentage of clinical recovery at follow-up suggests that DWM may be predictive of a good outcome, especially in NKH cases, although this needs to be confirmed in future studies.

## 1. Introduction

Peri-ictal brain imaging is increasingly recognized as a separate entity from structural and irreversible brain damage. Seizure-induced reversible MRI changes may appear within hours of seizure onset and resolve within a variable time frame, ranging from days to weeks [1,2,3]. Older brain neuroimaging techniques, made it harder to imagine reversible lesions, nonetheless reports of patients with seizure-related abnormalities appeared first on CT scans, as effacement of gyral marking and contrast enhancement [4]. Reversible lesions were increasingly reported [5], and many seem to be the result of prolonged disease activity, such as protracted seizures. Despite this, some studies reported possible irreversible damage following prolonged seizure activity [6], especially in terms of cerebral volume loss [7]. Many peri-ictal MRI abnormalities (PMAs) or seizure-induced reversible MRI abnormalities (SRMA) have been described: high- and low-intensity T2 signal abnormalities, diffusion-weighted imaging (DWI) changes, gadolinium enhancement, and the presence of single or multiple lesions involving transient abnormalities in the hippocampus, corpus callosum, and basal ganglia [8]. Among the best-known peri-ictal lesions are transient perfusion abnormalities such as focal hyperperfusion, which can be observed with arterial spin labeling (ASL) or perfusion MRI. These changes are particularly evident during seizure activity or shortly after [9]. The abnormalities are most frequently seen in T2-weighted and FLAIR sequences, and their presence seems to be most commonly encountered during status epilepticus (SE), favoring the hypothesis that prolonged seizure activity is required for their appearance [1]. For example, diffusion-restricted MRI lesions observed in the pulvinar of the thalamus and hippocampus are notoriously associated with status epilepticus [10]. Among these brain MRI reversible lesions, a recent systematic review by the author described the frequency of the so-called Dark White Matter—otherwise known as subcortical hypointensity on T2/FLAIR—in many diseases [11]. The most extensive case series on Dark White Matter included 29 patients, of whom 75.9% experienced seizures. Nearly half presented with associated extrinsic compressive lesions, most commonly subdural hematoma (SDH), which was observed in 41.8% of cases [12]. DWM is an interesting and frequently overlooked sign, with a possibly underestimated prevalence. In numerous published cases, diffuse subcortical white matter hypointensity is evident on MRI, yet it often goes unreported. This occurs, for instance, in reports of Myelin oligodendrocyte glycoprotein antibody-associated disease (MOGAD) encephalitis and status epilepticus, where dark white matter (DWM) appears in the imaging but is not mentioned in the figure legends, and is consequently overlooked [13,14,15,16]. Classical anatomical teachings focus on the cortex to explain the clinical manifestations of many diseases and seizures. Consequently, radiological and clinical focus has classically been on the cortex, and the white matter alterations are often underrecognized. DWM represents a decrease in signal intensity on T2/FLAIR MRI sequences that may appear isolated or associated with cortical changes. Its clinical relevance is particularly high in seizures, since its detection may suggest specific underlying etiologies such as NKH or MOGAD, reduce diagnostic delay in non-convulsive status epilepticus, and avoid unnecessary treatments (e.g., anti-seizure medications in glycemia-related seizures). Its recognition may thus impact both diagnosis and therapeutic decision-making. A recent systematic evidence by the authors [11], of more than 200 cases, showed that DWM is associated with seizures in over 60% of cases and is highly predictive of specific etiologies, primarily non-ketotic hyperglycemic hyperosmolar state (NKH). This sign is also found in Moyamoya disease, genetic causes, seizures related to encephalitis, and SDH. The most commonly encountered form of encephalitis associated with DWM is anti-MOG encephalitis [11]. Based on our previous work, we decided to reinterpret earlier findings with a particular focus on the presence of DWM in patients with seizures only. Features of patients presenting with seizures and DWM are discussed in light of the recent literature on the matter and peri-ictal imaging. This study builds upon our previously published systematic review, expanding on the semeiology of patients collected in our previous search and in light of new evidence. The present review aims further to characterize the DWM radiological sign in patients with seizures, re-analyzing and contextualizing previously reported data. We hypothesize that DWM may serve as a peri-ictal imaging marker indicative of transient subcortical network dysfunction. Its recognition could aid in etiological classification and guide clinical decision-making, particularly by supporting targeted therapy and avoiding unnecessary anti-seizure medications in NKH-related seizures, which often resolve with glycemic control. We aimed to better characterize NKH-related seizures from other etiologies, in the context of DWM. Furthermore, we suggest that DWM detection may facilitate the identification of other underlying etiologies associated with this imaging pattern.

## 2. Materials and Methods

PubMed was searched with the following main search string from the previous article with a specification to include patients with seizures only: (“Decreased” OR “Reduced” OR “Low Intensity” OR “Hypointensity”) AND (“Diffuse” OR “Subcortical” OR “White Matter” OR “Dark”) AND (“FLAIR” OR “T2” OR “T2WI” OR “Fluid-Attenuated Inversion Recovery” OR “T2-FLAIR”) AND (“Seizure” OR “Status Epilepticus”). The inclusion and exclusion criteria were those in our previous publication. A complementary search was performed from 1 September 2023, to 2 May 2025, to cover the missing period from our last search. A total of 208 records were screened, and none met the inclusion criteria from the PubMed search. Therefore, we assumed our previous search was still up-to-date, being the DWM sign a relatively rare one, and our last work, still the most comprehensive and the only systematic review on the literature so far on DWM [11]. Therefore, we used the previous supplementary database to analyze data of patients with seizures only, which was not the primary focus of the last review, to summarize the features of seizures associated with DWM. Moreover, we enriched the previous database with new and previously uncollected seizure information. Variables included are specified in the following paragraphs. The previous database was resized and filtered for seizures only. Data was analyzed using Excel and SPSS (release 26.0) to generate tables describing this subgroup of patients (DWM and Seizures). Our previous results were reinterpreted in light of new evidence. Missing data, lack of follow-up, and missing individual patient data were the main limitations of the study and the subsequent analysis.

We used the following Inclusion criteria: we included all the studies reporting Diffuse Subcortical White Matter Hypointensity. Non-English papers were also included. Articles included were only case reports and case series, as no evidence of higher quality is available. The main features extracted and systematically added in the database were: age, sex, clinical presentation, localization of the subcortical white matter hypointensity, presence of cortical abnormalities, contrast enhancement, primary diagnosis/underlying disorder, EEG, metabolic profile (glycaemia, HbA1c, osmolarity), advanced perfusion techniques, resolution of DWM, clinical recovery, follow-up MRI and mean resolution time when available. We also implemented information about history of previously diagnosed epilepsy, underlying etiology of epilepsy, seizure type, recurrence of seizures, clinical semiology of the seizure, type of EEG monitoring performed (continuous EEG vs. point-of-care or routine EEG), therapeutic interventions applied for seizure control, magnetic resonance imaging technical specifications, relevant medical or neurological comorbidities. Data on the duration and frequency of seizures, presence of substance use or abuse, the approximate duration of the ictal event, and the presence of drug-resistant epilepsy were largely unavailable across the included studies. This limited our ability to perform subgroup analyses or assess their potential association with the emergence or resolution of DWM abnormalities.

The following are the Exclusion criteria we used: intraparenchymal metastasis and intraparenchymal tumors were excluded as they do not result in subcortical alterations, but rather perilesional white matter alterations, which are out of the scope of the review; leptomeningeal metastasis and meningiomas were included. Basal ganglia hypointensities were excluded. We excluded patients already cited in other case series from the analysis to avoid duplication of cases. Case reports of MRI images where DWM was present but not described were not included in the database, as these are not searchable and were encountered by chance. First author (GM) performed the new research. Two expert neuroradiologists, ODB and ET, revised the image material. ADR performed statistical analysis. Every article was assessed individually, and every reference within articles was checked to exclude duplicate patients and to increase the identification of relevant studies.

The methodological quality of all included case reports was assessed using the Joanna Briggs Institute (JBI) Critical Appraisal Checklist for Case Reports [17], which comprises specific domains evaluating clarity in patient data reporting. A global risk of bias judgment was derived for each study based on the completeness and transparency of reporting, particularly regarding the presence or absence of adverse or unexpected events. Most reports were classified as moderate risk of bias. No reports were judged to be at high risk of bias. Still, the main limitation remains missing data.

Seizure types were reclassified according to the 2025 ILAE classification, based on seizure type (focal, generalized, unknown), predominant semiology (observable or not observable manifestations), and consciousness level (preserved or not) whenever possible [18]. The use of older classifications in the included reported cases is another recognized limitation.

## 3. Dark White Matter and Disease Patterns

Based on our previous systematic review, in the subgroup of patients presenting with seizures (a total of 140 patients), DWM was suggestive of NKH in 51.4% of cases, encephalitis in 26.4%, seizures alone in 7.8%, and SDH in 5.7% of cases [11]. Table 1 summarizes the demographic features of the 140 DWM and seizure patients.

**Figure 1:**
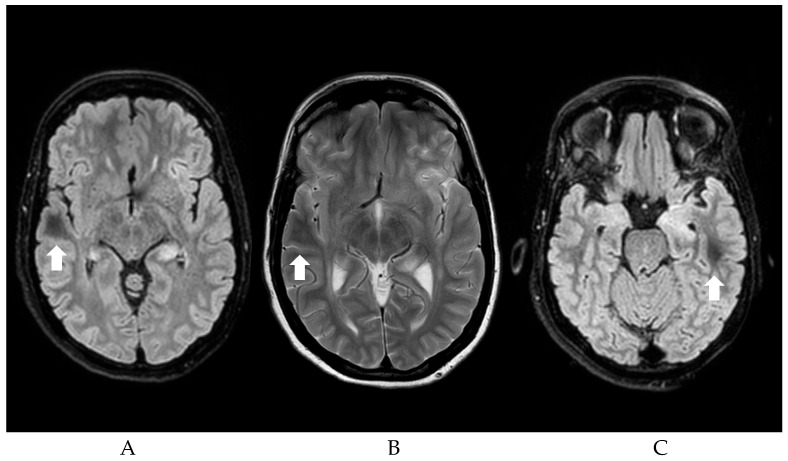
(**A**,**C**) FLAIR sequences showing Subcortical localization of DWM (Dark White Matter, see arrows) of a patient with NCSE; (**B**) T2 Sequences showing DWM.

Consequently, the most commonly described etiologies of DWM and seizures will be discussed here. Regardless of etiology, the occipital lobes seem to be the most common localization of DWM in patients with seizures (32.2%), followed by parietal (27.7%), frontal (21.9%), and temporal lobe (18%). Motor symptoms are the most commonly represented clinical manifestations (64.4%), followed by visual symptoms (37.5%). Figure 1 shows an example of the DWM.

### 3.1. Dark White Matter in Non-Ketotic Hyperglycemic Hyperosmolar State

Among the two distinct forms of hyperglycemia—ketotic and non-ketotic—seizures are more frequently associated with NKH [19,20]. Previous studies and case reports involving patients with mildly elevated serum osmolarity suggest that seizures are more likely triggered by chronic hyperglycemia associated with abnormal HbA1c levels, rather than by an acute hyperosmolar hyperglycemic state [21,22]. Notably, the high reversibility rate of these changes reflects their transient nature and tendency to resolve completely with normalization of blood glucose levels, reflecting the functional rather than structural nature of the underlying pathophysiology. Diabetic patients with poor glycemic control (HbA1c > 74 mmol/mol) exhibit a significantly increased risk of seizures. Seizures induced by hyperglycemia are typically resistant to anti-seizure medications unless blood glucose levels are adequately managed [23,24]. Previous literature suggested T2/FLAIR subcortical hypointensity (DWM) as a main neuroradiological hallmark of NKH-induced seizures [25,26]. A recent systematic review on NKH-associated seizure showed that 48.7% of cases presented focal T2 subcortical hypointensity and/or overlying cortical T2 hyperintensity with DWI restriction [27].

Notably, from our previous literature review, clear evidence of seizures was present in almost all cases of NKH, with the occipital lobe being the most commonly affected region (54.9%), and unilateral involvement was observed in every case of NKH patients with seizures. Seizures were almost exclusively focal. The most frequently reported clinical manifestations include observable manifestations, such as focal motor seizures (54.1%), and non-observable manifestations, such as visual symptoms (52.7%), with hemianopia and visual hallucinations being the most prevalent among the latter. DWM is reversible in 86.3% of NKH cases with follow-up MRI data. Cortical abnormalities were identified in 57% of cases, and cortical enhancement, when contrast was administered, was reported in 40%. The mean glucose level in NKH patients was 458.8 mg/dL, with average HbA1c and serum osmolarity values of 117.8 mmol/mol and 305.1 mosm/L, respectively [11]. Table 2 summarizes the clinical features of NKH and DWM patients.

It is interesting to note how the percentage of patients with occipital involvement and visual symptoms increases when considering only the patients with NKH. Occipital lobe involvement seems to be a distinguishing feature of seizures during NKH. Isolated DWM without cortical involvement was seen in 43% of patients. Interestingly, in NKH patients, the mean lesion resolution time was substantially shorter in those with cortical abnormalities (85.8 vs. 199.7 days); however, this difference was not statistically significant (*t*-test: *t* = 1.30, *p* = 0.213), likely due to small sample size. Considering the subgroup of NKH patients, these patients exhibited almost exclusively focal seizures (motor or visual), visual symptoms were reported in more than half of the NKH patients, with homonymous hemianopia and hallucination being the most prevalent findings, followed by nonspecific visual disturbances such as blurred vision.

The duration of visual symptoms in patients with NKH was significantly longer than that of motor symptoms, suggesting a potentially more prolonged postictal course or lesional involvement; specifically, visual symptoms had a mean duration of 9.13 days versus 4.40 days for motor symptoms (unpaired *t*-test with unequal variances; *t* = 2.08; *p* = 0.049).

These findings suggest a predominant involvement of the occipital lobe and visual pathways, supporting the hypothesis that DWM reflects transient dysfunction in metabolically vulnerable posterior cortical regions. Moreover, all patients responded to glycaemic control, but 25 required anti-seizure medication, with reported later suspension in four.

### 3.2. Dark White Matter in MOG-Encephalitis, Subdural Hematoma and Other Etiologies

DWM was found in 9 (23.7%) of 38 patients with encephalitis (mostly viral: two herpetic, seven non-herpetic), 5 (24%) of 21 with leptomeningeal metastasis, and 5 (9%) of 58 with meningitis, in a previous report [28]. The authors reported that subcortical low intensity disappeared or decreased in extent on follow-up MR images in all seven patients who underwent follow-up [28]. In encephalitis, the second most frequent condition linked to DWM in our previous work, seizures appeared to be the predominant manifestation. However, data on lobar involvement and a clearer specification were mostly lacking. In 100% of these cases with encephalitis and seizures, the objective of this review, the underlying etiology was MOGAD [11]. MOG-encephalitis seems to be the most commonly associated encephalitis with DWM in our review. In the most extensive case series, 95% of MOG-encephalitis patients presented with DWM adjacent to the cortical lesions [29]. All these patients had unilateral cerebral cortical T2 FLAIR hyperintense lesions on MRI; T2-FLAIR hypointensity in the subcortical white matter is a characteristic feature of anti-MOG antibody-associated unilateral cerebral cortical encephalitis, and may develop as a secondary effect of cortical involvement in this setting. The coexistence of cortical T2-FLAIR hyperintensities with adjacent subcortical T2-FLAIR hypointensities could represent this condition’s imaging distinctive pattern [29].

Negative vascular symptoms were the primary clinical features of the third most common DWM-associated condition, Moyamoya disease. No patients reported seizures in this cohort.

As shown in our previous work, SDH is the fourth most common condition associated with DWM. On the contrary, the most extensive case series of DWM reported it to be the most prevalent one in patients with seizures [12]. In our previous work, among patients with seizures and DWM, 8 had SDH, suggesting that DWM may also occur in the context of compressive or hemorrhagic etiologies and is not limited to metabolic or autoimmune processes; of these 8 SDH patients, three were post-operative; all had focal motor seizures [11].

A broad spectrum of etiologies was observed among less frequently reported cases of DWM and seizures. These included: tumors (3), cardiac arrest (2), subarachnoid hemorrhage (SAH, 2), and genetic causes (2). Additionally, less frequent causes included post-embolization venous thrombosis after dural arteriovenous fistula, bacterial meningitis, traumatic brain injury, and multiple sclerosis. These cases underscore the potential of DWM to emerge in various settings of acute structural, infectious, and post-operative brain insults, where transient subcortical perfusion or metabolic disruption may occur.

### 3.3. Generalized Versus Focal Seizures

Cortical abnormalities were exclusively observed in patients with focal seizures, among patients for whom seizure type information was available. Cortical abnormalities were more frequent among patients who achieved full imaging recovery (40 with cortical vs. 23 without), suggesting a potential association between cortical involvement and favorable outcome. Focal or epileptiform EEG abnormalities were reported in 56 patients. In comparison, diffuse slowing was reported in 9 patients, reinforcing the potential link between DWM and epileptogenic activity or seizure propagation pathways. Contrast enhancement on MRI was observed in 30 patients (29%), which may reflect underlying inflammation, blood–brain barrier disruption, or vasogenic changes. Perfusion imaging (e.g., SPECT or ASL) was only available in 13 patients, and 7 showed increased perfusion. In these cases, hyperperfusion was located within the same region as the DWM signal abnormalities. A higher proportion of patients with cortical abnormalities showed perfusion increase compared to those without; however, this association did not reach statistical significance (odds ratio = 6; probability odds ratio *p* = 0.86), likely due to the small sample size. Still, the findings are particularly relevant as perfusion alterations support the hypothesis of a reversible reperfusion-related mechanism contributing to DWM. These findings indicate that DWM is rarely an isolated phenomenon; instead, it tends to occur in a broader pathological process involving both cortical and subcortical regions, with functional and structural changes detectable across multiple modalities. The most frequently concomitant cortical abnormalities associated with DWM are diffusion restriction (DWI was positive in 27 patients), T2 and FLAIR cortical hyperintensities, respectively reported in 25 and 24 patients. Interestingly, DWM-related seizures are mainly focal (see Table 3).

Table 3 summarizes key clinical and diagnostic features across the cohort. The majority of the DWM-related seizures are focal. A known diagnosis of epilepsy was present in 12 cases. Seizure semiology is provided in the Table. Repetitive/cluster seizures were present in 35 patients (25%). Data regarding seizure duration was mostly unavailable; in NKH patients, the symptom persistence was 7.64 days. SE occurred in 13 patients (9.3%), including 11 convulsive (5 of those with epileptic partialis continua (EPC)), 2 non-convulsive. ASMs were administered in 28 cases and were discontinued at follow-up, mainly in NKH. The most frequent comorbidity was diabetes (40.7%), followed by hypertension (12.1%) and chronic kidney disease (2.9%).

### 3.4. Follow-Up and Evolution

Overall clinical recovery was observed in 103 patients (73.6%). However, in many cases, follow-up is missing. Only two instances report no clinical improvement, probably due to the extended follow-up required and to the underlying pathological process.

Among the 41 patients for whom DWM resolution time was available, the median time to resolution was 60 days. At the same time, the mean duration was approximately 184 days, indicating a positively skewed distribution with some prolonged cases.

These findings suggest that although DWM often resolves within two to three months, in a subset of patients it can persist long-term, possibly reflecting ongoing subcortical dysfunction or underlying structural damage.

The mean duration of DWM resolution was shorter in NKH patients (136.1 days) compared to non-NKH patients (477 days); however, this difference did not reach statistical significance (*p* = 0.37; unpaired *t*-test with unequal variances, *t* = −0.97).

## 4. Proposed Pathophysiology

The pathogenesis of DWM remains hypothetical and subject to ongoing debate. Several mechanisms have been proposed to explain the observed T2-FLAIR hypointensity, including T2 shortening due to increased protein content, high cellularity, demyelination, calcium deposition, heme and non-heme iron accumulation, and free radicals [30]. The leading theory suggests that transient subcortical deposits of non-heme iron arise from interrupted axonal transport and oxidative stress after excitotoxic axonal injury during seizures [22,31]. Although DWM is reversible in many cases, reversibility on imaging does not always imply complete histopathological recovery. Autopsy studies have occasionally shown pallor of the white matter in areas corresponding to DWM, suggesting residual structural changes [31]. In instances of spontaneous intracranial hypotension and leptomeningeal metastases, elevated deoxyhemoglobin due to venous stasis has been proposed as an additional potential cause of DWM [32], and a similar mechanism may apply to extrinsic compressive lesions such as subdural hematomas or metastatic infiltration [12]. Among the numerous hypotheses, the accumulation of free radicals is among the most credible. We endorse that oxidative stress is pivotal, given that many conditions linked to DWM seem to converge on a common pathogenic mechanism. In our earlier publication, we proposed a biphasic model of hypo-hyperperfusion. We suggest that during seizures, the hyperperfusion phase is likely dominant. Hyperperfusion in the context of seizures has been noted since the initial observations by Gibbs and Penfield [33,34]. In this model, we previously suggested that the initial phase—impaired perfusion—initiates a gradual hypoxic-ischemic state instead of an acute event. This could cause a transition from aerobic to anaerobic metabolism, as observed in NKH due to microvascular damage and hyperglycemia [31], in subdural hematoma due to mechanical compression, and in MOGAD to a condition called virtual hypoxia, recently recognized in autoimmune neurological disorders [35]. Chronic hyperglycemia may render subcortical white matter and the underlying cortex more susceptible to seizures; indeed, seizures appear to happen in a later stage in NKH. The subsequent phase is characterized by hyperperfusion, which has been observed during seizures [9], NKH-related seizures [36,37], and after evacuation of subdural hematomas [38]. In 7 of 14 cases in which perfusion imaging was available, hyperperfusion was observed. This reperfusion phase may promote the production of free radicals and inflammatory mediators, potentially explaining the T2 shortening observed in DWM. Future studies are needed to better elucidate DWM pathogenesis. In particular, the limited use of advanced perfusion techniques in the existing literature underscores the need for prospective studies incorporating SPECT, ASL, and other modalities to elucidate the hemodynamic profile of DWM better.

## 5. Discussion

Many studies have reported numerous peri-ictal MRI abnormalities, including increased DWI with apparent diffusion coefficient modifications and increased T2 and FLAIR signal [39]. A posterior overrepresentation has been suggested in the literature for PMAs [39], which seems to be shared by DWM too: the occipital lobe is the most frequently involved, possibly due to lower autonomic regulation. PMAs’ prevalence varies greatly and is likely observed with longer seizure duration [40,41,42]. Moreover, PMAs were more frequently associated with refractory and super-refractory SE; in a multivariate analysis, PMAs were associated with SE duration [3]. The mean resolution time of DWM seems to be longer than previously reported in the literature in other case series, such as those by Mariajoseph et al. (183.6 versus 96.5 days) [1].

Reduced signal in the subcortical white matter on T2-weighted and FLAIR MRI has been observed in a variety of clinical scenarios, including intracranial hypotension, infections like encephalitis and meningitis, head trauma, leptomeningeal spread of disease, diffuse axonal injury, cortical ischemia, and seizures occurring in the setting of nonketotic hyperglycemic hyperosmolar states [12]. One of the most extensive case series on DWM and seizures reported their presence mostly in SDH, with a clinical, electroencephalographic, and anatomical correlate on EEG and brain MRI [12]. When present, DWM should be promptly recognized due to its diagnostic significance, particularly as a potential clue to NKH in seizures with unclear etiology. Seizures related to NKH often respond well to correction of blood glucose levels but are typically resistant to standard anti-seizure medications [43].

The elevated prevalence of cortical and focal abnormalities, on MRI and EEG, respectively, reinforces the potential link between DWM and epileptogenic activity or seizure propagation pathways. Observed contrast enhancement on MRI may reflect underlying inflammation, blood–brain barrier disruption, or vasogenic changes. Perfusion imaging (e.g., SPECT or ASL) was only available in 14 patients. Still, the findings are relevant: perfusion alterations further support the hypothesis of a reversible reperfusion-related mechanism contributing to DWM.

Diffuse subcortical white matter hypointensity on FLAIR/T2 (DWM) in patients with seizures represents a radiological finding whose pathophysiological significance remains poorly understood. In this series, which specifically included patients with seizures and DWM, we observed a spectrum of clinical presentations and etiologies, suggesting that DWM is not associated with a single pathogenic mechanism but rather represents a final typical radiological phenotype shared across diverse conditions. From a pathophysiological standpoint, our findings support the hypothesis that DWM reflects subcortical tissue vulnerability, particularly in long-standing hyperglycemia. Conversely, in inflammatory or autoimmune etiologies, such as MOGAD or encephalitis, DWM may reflect transient demyelination, vasogenic edema, or immune-mediated dysfunction, which are potentially reversible under appropriate treatment, an interpretation supported by the favorable outcomes seen in these patients. The post-surgical and post-traumatic subgroup, including cases with SDH, exhibited intermediate characteristics. In these patients, DWM may arise from venous congestion, altered perfusion, or mechanical compression, potentially compounded by post-ictal changes. This group likely reflects a combination of transient and permanent structural damage.

Importantly, DWM was more frequently associated with non-generalized seizures and with cortical abnormalities on MRI, suggesting a possible interplay between cortical and subcortical structures in epileptogenesis. Interestingly, in NKH patients, the mean lesion resolution time was substantially shorter in those with cortical abnormalities (85.8 vs. 199.7 days); however, this difference was not statistically significant. This tendency may be explained by subcortical abnormalities being more related to long-standing hyperglycemia, leading to disruption of the axonal subcortical transportation network. However, the duration of seizures and hyperglycemia is not available in the analyzed case reports; future studies are needed to clarify this point. DWM is not solely found in the context of seizure. Nonetheless, its presence in the context of seizures can aid in the diagnostic process. As already discussed, important clues exist towards etiologies such as NKH, anti-MOG encephalitis, and SDH. Moreover, we previously reported the diagnostic value of DWM in non-convulsive status epilepticus (NCSE), especially when Salzburg criteria [44] are not met and diagnostic doubt persists; the presence of DWM, when found, can aid in the diagnosis of NCSE, with significant implications on treatment and the patient outcome [45].

The present data provide novel insights into the clinical and radiological significance of the DWM sign in patients with seizures. One of the most compelling findings is the high proportion of patients achieving clinical recovery (73.6%), despite the frequent occurrence of repetitive seizures and status epilepticus. This supports the hypothesis that DWM may not only serve as a peri-ictal diagnostic marker but also as an indicator of favorable prognosis in most cases. This may contrast with other PMAs, such as cortical diffusion abnormalities, which have been linked to poor outcomes [3]. The high reversibility and recovery rates suggest DWM may reflect a functional marker rather than irreversible damage. However, this hypothesis must be interpreted cautiously and addressed in future studies.

An interesting differential pattern also emerges when analyzing symptom duration by semiology. Cases with visual symptoms, often presenting as hemianopia or visual hallucinations, showed longer persistence of symptoms compared to those with motor manifestations. This disparity may reflect underlying pathophysiological differences: hemianopia, typically a negative symptom, may reflect more structural, still reversible, damage rather than an ictal phenomenon per se. In contrast, visual hallucinations, being positive symptoms, are more likely to reflect critical ictal activity and often accompany negative ones. This may also reflect a higher vulnerability of the posterior cortical and subcortical regions.

The frequent observation of repetitive seizures (present in over 25% of patients) and SE (present in 9.2%) further suggests that DWM may be more likely to emerge in the context of prolonged or recurrent ictal activity. This is also shown in NKH patients with visual persistence of symptoms over many days. Although precise timing was not always available, this aligns with literature on other peri-ictal MRI changes, such as transient cortical hyperintensities or diffusion restriction, which are more common in prolonged critical states. DWM may thus represent a form of subcortical correlates of cortical hypermetabolism, particularly when ictal activity is sustained or poorly controlled. DWM should be re-evaluated not merely as a subtle or under-recognized imaging sign, but as a clinically meaningful biomarker of active disease. Its presence may offer diagnostic support, particularly in ambiguous or EEG-negative cases, and may also provide prognostic reassurance when recognized in the appropriate clinical and temporal context. DWM is also present in convulsive SE, including EPC. This broadens the potential diagnostic value of DWM across the entire status epilepticus spectrum, supporting its consideration in convulsive and non-convulsive settings.

This study has several limitations: the retrospective nature of most included reports resulted in variable data quality. Outcome measures were inconsistently reported, precluding firm conclusions on the prognostic relevance of DWM. Moreover, little information about patient history, seizure duration, and semeiology is available. The old ILAE classifications of seizures were used in different studies, limiting the proper classification of seizures. Whenever possible, we re-interpreted the seizure semeiology in light of the most recent classification of seizure ILAE 2025 [18]. Selection bias is likely, as unusual imaging findings are more often reported. Furthermore, the absence of systematic EEG or imaging protocols in many cases limits generalizability.

A key limitation is the inability to disentangle whether DWM results from seizure activity or reflects the underlying disease (e.g., NKH or MOGAD). In prospective studies, this issue should be addressed with serial imaging and standardized protocols. Nonetheless, the author believes that DWM is only partially etiology-specific. This observation stems from the higher prevalence and more precise imaging delineation of DWM in cases of non-ketotic hyperglycemia, where the subcortical hypointensity is often more conspicuous and radiologically striking. Future studies should aim to explore its occurrence in diverse pathological contexts, ideally incorporating quantitative measures of subcortical T2/FLAIR hypointensity to improve detection and comparability.

Overall, these observations suggest that DWM in seizure patients is a heterogeneous and partially etiology-dependent phenomenon, with implications for both prognosis and pathophysiological interpretation. Recognition of the underlying context in which DWM occurs is essential to avoid diagnostic oversight and to tailor appropriate therapeutic strategies.

## 6. Conclusions

Dark White Matter can be a peri-ictal MRI sign; its significance is diagnostic and prognostic. Clinicians should be familiar with it, as it may aid in the diagnostic process. As already shown when DWM happens in isolation, without cortical involvement, this pattern is more suggestive of NKH. On the other hand, when overlying cortical abnormalities are also present, MOGAD should be kept in mind as the more likely underlying disorder. Moreover, DWM and other PMAs can aid in diagnosing NCSE and convulsive SE. Future studies are needed to confirm the presence of DWM in NCSE, its clinical relevance in different settings, and whether it is present in other forms of encephalitis, besides the anti-MOG form. The identification of DWM, as well as other PMAs, can help guide therapeutic interventions and orient a diagnosis of epileptic disorder in challenging cases. Moreover, knowledge and recognition of PMAs prevent unnecessary investigations such as brain biopsy. Prospective studies that further investigate the pathophysiology, primarily through advanced neuroimaging techniques and molecular biomarkers, are needed to characterize and quantify DWM.

## Figures and Tables

**Table 1:** Demographic features of patients with seizures and DWM.

Variable	Value
Total number of patients	140
Mean age (years)	53.1
Mean imaging resolution time (days)	183.6
Eziologies	
NKH	72 (51.4%)
MOGAD-Encephalitis	37 (26.4%)
Seizures alone	11 (7.8%)
SDH	8 (5.7%)
Other	12 (8.6%)

DWM = Dark White Matter. NKH = Non-Ketotic Hyperglycemic Hyperosmolar State. SDH = subdural hematoma. MOGAD = Myelin oligodendrocyte glycoprotein (MOG) antibody-associated disease.

**Table 2:** Demographic and clinical-radiological characteristics of Non-Ketotic hyperglycaemic Hyperosmolar syndrome (NKH) patients.

	Mean or Number of Patients	Standard Deviation or Percentage (%)	Available/Calculated on (Number of Patients) of the Total	
Age	56.8	13.0	72	of 72
Sex	40 (M)	28 (F)	68	of 72
DWM Localization				
Frontal	16	22.5%	71	of 72
Temporal	15	21.1%		
Parietal	28	39.4%		
Occipital	39	54.9%		
Unilateral	71	100%	72	of 72
Bilateral	0	0%		
Follow-up imaging Resolved in	38	86.3%	44	of 72
Mean Res Time (days)	136	227.2		
EEG				
EEG focal	43	75.4%	57	of 72
EEG diffuse	4	7%		
EEG normal	10	17.5%		
ASM Used	25	(4 suspended)		
Metabolic Profile				
Glycemia (mg/dl)	458.8	126.7	72	of 72
HbA1c (mmol/mol)	117.8	46.5	50	of 72
Osmolarity (osm/l)	305.1	11	54	of 72
Seizure Classification			72	of 72
Focal seizures	68			
Generalized	1			
Unknown	3			
Clinical Manifestations				
Not Observable (Visual)	38	52.7%		
Hallucination	20			
Hemianopia	20			
Observable (Motor)	39	54.1%		
Other	5	7%		
Symptoms Persistaence (days)	7.64			
Associated Seizures	72	100%	72	of 72
No Cortical Involvement	31	43%		
Cortical Enhancement	29	40%		
Negative T2 Shine Through ADC	3			
Hyper perfusion (SPECT/ASL)	5	45.5%	11	of 72

Abbreviations: DWM = Dark White Matter; NKH = Non-ketotic hyperglycemia; SE = Status Epilepticus; ASL = Arterial Spin Labeling; SPECT = Single Photon Emission Computed Tomography.

**Table 3:** Key Semiological features of the entire population.

	Number of Patients	
Known Epilepsy	12 (8.65%)	
Structural etiology (tumors)	3	
Seizure Classification		
Focal	87 (62%)	
Focal to Bilateral tonic-clonic seizure	11	
Focal preserved consciousness seizure	5	
Focal impaired consciousness seizure	11	
Generalized	16 (11.4%)	
GTCS	6	
Unknown	37	
Repetitive Seizures (more than 1)	35 (25%)	8.6 on average
Status Epilepticus (11 Convulsive)	13 (9.3%)	
Status Epilepticus (EPC)	5	
EEG		
POC-EEG	81	
C-EEG	6	
Focal	56	
Diffuse	9	
ASM Used	28	
Clinical Recovery	103 (73.6%)	
Comorbidities		
Diabetes	57 (40.7%)	
Hypertension	17 (12.1%)	
CKD	4 (2.9%)	
Other	<3%	
MRI		
1.5T	47	
3T	2	
0.4T	1	
Contrast Enhancement	30	
Cortical Involvement (unspecified)	45	
Cortical T2/FLAIR involvement	25/24	
Cortical DWI involvement	27	
Hyperperfusion (SPECT/ASL)	7	

Abbreviations: ASM = Anti-Seizure Medication, C-EEG = Continuous Electroencephalogram, CKD = Chronic Kidney Disease, DWM = Dark White Matter, EPC = Epilepsia Partialis Continua, GTCS = Generalized Tonic-Clonic Seizures, MRI = Magnetic Resonance Imaging, POC-EEG = Point-of-Care Electroencephalogram, SE = Status Epilepticus, T = Tesla.

## Data Availability

Not applicable.

## Abbreviations

The following abbreviations are used in this manuscript:

DWM	Dark White Matter—Subcortical Low Intensity on T2/FLAIR
SE	Status Epilepticus
NKH	Non-Ketotic Hyperglycaemic hyperosmolar State
SDH	SubDural Hematoma
SRMA	Seizure-induced reversible MRI abnormalities
PMAs	Peri-ictal MRI abnormalities
ASL	Arterial Spin Labeling;
EEG	Electroencephalogram
C-EEG	Continuous EEG monitoring
POC-EEG	Point-of-care EEG
ASM	Anti-seizure medication
MRI	Magnetic Resonance Imaging
FLAIR	Fluid-attenuated inversion recovery
DWI	Diffusion-Weighted Imaging
SDH	Subdural Hematoma
MOGAD	Myelin oligodendrocyte glycoprotein antibody-associated disease
GTCS	Generalized Tonic-Clonic Seizures
EPC	Epilepsia Partialis Continua
CKD	Chronic Kidney Disease

## References

[1] Mariajoseph F.P., Sagar P., Muthusamy S., Amukotuwa S., Seneviratne U. (2021). Seizure-induced reversible MRI abnormalities in status epilepticus: A systematic review. Seizure.

[2] Pascarella A., Manzo L., Marsico O., Africa E., Coglitore A., Cianci V., Bulgari A., Abelardo D., Gasparini S., Armentano A. (2025). Investigating Peri-Ictal MRI Abnormalities: A Prospective Neuroimaging Study on Status Epilepticus, Seizure Clusters, and Single Seizures. J. Clin. Med..

[3] Bonduelle T., Ollivier M., Trin K., Thomas B., Daubigney A., Michel V., De Montaudouin M., Marchal C., Aupy J. (2023). Association of Peri-ictal MRI Abnormalities with Mortality, Antiseizure Medication Refractoriness, and Morbidity in Status Epilepticus. Neurology.

[4] Goulatia R.K., Verma A., Mishra N.K., Ahuja G.K. (1987). Disappearing CT lesions in epilepsy. Epilepsia.

[5] Cole A.J. (2004). Status epilepticus and periictal imaging. Epilepsia.

[6] Canas N., Breia P., Soares P., Saraiva P., Calado S., Jordão C., Vale J. (2010). The electroclinical-imagiological spectrum and long-term outcome of transient periictal MRI abnormalities. Epilepsy Res..

[7] Bosque Varela P., Machegger L., Steinbacher J., Oellerer A., Pfaff J., McCoy M., Trinka E., Kuchukhidze G. (2024). Brain damage caused by status epilepticus: A prospective MRI study. Epilepsy Behav..

[8] Cianfoni A., Caulo M., Cerase A., Della Marca G., Falcone C., Di Lella G.M., Gaudino S., Edwards J., Colosimo C. (2013). Seizure-induced brain lesions: A wide spectrum of variably reversible MRI abnormalities. Eur. J. Radiol..

[9] Schertz M., Benzakoun J., Pyatigorskaya N., Belkacem S., Sahli-Amor M., Navarro V., Cholet C., Leclercq D., Dormont D., Law-Ye B. (2020). Specificities of arterial spin labeling (ASL) abnormalities in acute seizure. J. Neuroradiol..

[10] Bosque Varela P., Tabaee Damavandi P., Machegger L., Prüwasser T., Zimmermann G., Oellerer A., Steinbacher J., McCoy M., Pfaff J., Trinka E. (2024). Magnetic resonance imaging fingerprints of status epilepticus: A case-control study. Epilepsia.

[11] Magro G., Tosto F., Laterza V., Di Benedetto O. (2024). The Dark side of the White Matter. Diffuse subcortical White Matter Hypointensity on T2/FLAIR: A systematic review of a frequently underrecognized sign. J. Neurol. Sci..

[12] Nicholson P., Abdulla S., Alshafai L., Mandell D.M., Krings T. (2020). Decreased Subcortical T2 FLAIR Signal Associated with Seizures. AJNR Am. J. Neuroradiol..

[13] Budhram A., Mirian A., Le C., Hosseini-Moghaddam S.M., Sharma M., Nicolle M.W. (2019). Unilateral cortical FLAIR-hyperintense Lesions in Anti-MOG-associated Encephalitis with Seizures (FLAMES): Characterization of a distinct clinico-radiographic syndrome. J. Neurol..

[14] Patterson K., Iglesias E., Nasrallah M., González-Álvarez V., Suñol M., Anton J., Saiz A., Lancaster E., Armangue T. (2019). Anti-MOG encephalitis mimicking small vessel CNS vasculitis. Neurol.-Neuroimmunol. Neuroinflamm..

[15] Wang L., ZhangBao J., Zhou L., Zhang Y., Li H., Li Y., Huang Y., Wang M., Lu C., Lu J. (2019). Encephalitis is an important clinical component of myelin oligodendrocyte glycoprotein antibody associated demyelination: A single-center cohort study in Shanghai, China. Eur. J. Neurol..

[16] Huang Y.C., Weng H.H., Tsai Y.T., Huang Y.C., Hsiao M.C., Wu C.Y., Lin Y.H., Hsu H.L., Lee J.D. (2009). Periictal magnetic resonance imaging in status epilepticus. Epilepsy Res..

[17] Munn Z., Barker T.H., Moola S., Tufanaru C., Stern C., McArthur A., Stephenson M., Aromataris E. (2020). Methodological quality of case series studies: An introduction to the JBI critical appraisal tool. JBI Evid. Synth..

[18] Beniczky S., Trinka E., Wirrell E., Abdulla F., Al Baradie R., Alonso Vanegas M., Auvin S., Singh M.B., Blumenfeld H., Bogacz Fressola A. (2025). Updated classification of epileptic seizures: Position paper of the International League Against Epilepsy. Epilepsia.

[19] Moien-Afshari F., Téllez-Zenteno J.F. (2009). Occipital seizures induced by hyperglycemia: A case report and review of literature. Seizure.

[20] Maccario M., Messis C.P., Vastola E.F. (1965). Focal Seizures as a Manifestation of Hyperglycemia Without Ketoacidosis. A Report of Seven Cases with Review of the Literature. Neurology.

[21] Hung W.L., Hsieh P.F., Lee Y.C., Chang M.H. (2010). Occipital lobe seizures related to marked elevation of hemoglobin A1C: Report of two cases. Seizure.

[22] Sasaki F., Kawajiri S., Nakajima S., Yamaguchi A., Tomizawa Y., Noda K., Hattori N., Okuma Y. (2016). Occipital lobe seizures and subcortical T2 and T2 * hypointensity associated with nonketotic hyperglycemia: A case report. J. Med. Case Rep..

[23] Pro S., Randi F., Pulitano P., Vicenzini E., Mecarelli O. (2011). Non-convulsive status epilepticus characterised exclusively by a language disorder induced by non-ketotic hyperglycaemia. Epileptic Disord..

[24] Lee E.J., Kim K.K., Lee E.K., Lee J.E. (2016). Characteristic MRI findings in hyperglycaemia-induced seizures: Diagnostic value of contrast-enhanced fluid-attenuated inversion recovery imaging. Clin. Radiol.

[25] De Martino S.R.M., Toni F., Spinardi L., Cirillo L. (2020). Magnetic resonance imaging findings in patients with non-ketotic hyperglycaemia and focal seizures. Neuroradiol. J..

[26] Raghavendra S., Ashalatha R., Thomas S.V., Kesavadas C. (2007). Focal neuronal loss, reversible subcortical focal T2 hypointensity in seizures with a nonketotic hyperglycemic hyperosmolar state. Neuroradiology.

[27] Licchetta L., Ferri L., Morsillo F., Faustini-Fustini M., Toni F., Pondrelli F., Nonino F., Bisulli F., Tinuper P. (2023). Clinical characterization of non-ketotic hyperglycemia-related seizures: A systematic review and individual participant data meta-analysis. Seizure.

[28] Lee J.H., Na D.G., Choi K.H., Kim K.J., Ryoo J.W., Lee S.Y., Suh Y.L. (2002). Subcortical low intensity on MR images of meningitis, viral encephalitis, and leptomeningeal metastasis. AJNR Am. J. Neuroradiol..

[29] Fujimori J., Ogawa R., Murata T., Takai Y., Misu T., Nakashima I. (2022). Decreased subcortical T2 FLAIR signal with cortical T2 FLAIR hyperintense lesions in unilateral cerebral cortical encephalitis with myelin oligodendrocyte glycoprotein antibody. Neuroimmunol. Rep..

[30] Cerase A., Leonini S., Franceschini R., Grosso S., Venturi C. (2011). Subcortical low-intensity and restricted diffusion after first seizure in a child. J. Comput. Assist. Tomogr..

[31] Seo D.W., Na D.G., Na D.L., Moon S.Y., Hong S.B. (2003). Subcortical hypointensity in partial status epilepticus associated with nonketotic hyperglycemia. J. Neuroimag..

[32] Adachi M., Mugikura S., Shibata A., Kawaguchi E., Sato T., Takahashi S. (2009). Relative decrease in signal intensity of subcortical white matter in spontaneous intracranial hypotension on fluid-attenuated inversion recovery images. AJNR Am. J. Neuroradiol..

[33] Gibbs F.A., Lennox W.G., Gibbs E.L. (1934). Cerebral Blood Flow Preceding and Accompanying Epileptic Seizures in Man. Arch. Neurol. Psychiatry.

[34] Penfield W. (1933). The Evidence for a Cerebral Vascular Mechanism in Epilepsy. Ann. Intern. Med..

[35] Lassmann H. (2022). The contribution of neuropathology to multiple sclerosis research. Eur. J. Neurol..

[36] Sekar S., Vinayagamani S., Thomas B., Kesavadas C. (2021). Arterial spin labeling hyperperfusion in seizures associated with non-ketotic hyperglycaemia: Is it merely a post-ictal phenomenon?. Neurol. Sci..

[37] Kobayashi Y., Itabashi R., Saito T., Kawabata Y., Yazawa Y. (2021). Irreversible Homonymous Hemianopia Associated with Severe Hyperglycemia and Cerebral Hyperperfusion: A Case Report and Literature Review. Intern. Med..

[38] Katsuki M., Narita N., Watanabe O., Cai S., Ishida N., Tominaga T. (2021). Endoscopically Treated Subacute Subdural Hematoma Presenting Postoperative Cerebral Hyperperfusion Syndrome: Chronological Changes of Cerebral Blood Flow on Arterial Spin Labeling and Subcortical Low Intensity on Fluid-attenuated Inversion Recovery Images. NMC Case Rep. J..

[39] Williams J.A., Bede P., Doherty C.P. (2017). An exploration of the spectrum of peri-ictal MRI change; a comprehensive literature review. Seizure.

[40] Giovannini G., Kuchukhidze G., McCoy M.R., Meletti S., Trinka E. (2018). Neuroimaging alterations related to status epilepticus in an adult population: Definition of MRI findings and clinical-EEG correlation. Epilepsia.

[41] Jabeen S.A., Cherukuri P., Mridula R., Harshavardhana K.R., Gaddamanugu P., Sarva S., Meena A.K., Borgohain R., Jyotsna Rani Y. (2017). A prospective study of diffusion weighted magnetic resonance imaging abnormalities in patients with cluster of seizures and status epilepticus. Clin. Neurol. Neurosurg..

[42] Requena M., Sarria-Estrada S., Santamarina E., Quintana M., Sueiras M., Rovira A., Toledo M. (2019). Peri-ictal magnetic resonance imaging in status epilepticus: Temporal relationship and prognostic value in 60 patients. Seizure.

[43] Paoletti M., Bacila A., Pichiecchio A., Farina L.M., Rognone E., Cremascoli R., Fanucchi S., Manni R., Bastianello S. (2018). Atypical postictal transient subcortical T2 hypointensity in a newly diagnosed diabetic patient with seizures. Epileptic Disord..

[44] Leitinger M., Trinka E., Zimmermann G., Beniczky S. (2019). Salzburg criteria for nonconvulsive status epilepticus: Details matter. Epilepsia.

[45] Magro G., Di Benedetto O., Laterza V., Tosto F. (2024). Reversible subcortical Dark White Matter lesions in nonconvulsive status epilepticus: A rare but clinically significant finding. Acta Neurol. Belg..

